# Bcl-x_L_ is an oncogenic driver in colorectal cancer

**DOI:** 10.1038/cddis.2016.233

**Published:** 2016-08-18

**Authors:** Anna-Lena Scherr, Georg Gdynia, Mariam Salou, Praveen Radhakrishnan, Katarina Duglova, Anette Heller, Sophia Keim, Nicole Kautz, Adam Jassowicz, Christin Elssner, You-Wen He, Dirk Jaeger, Mathias Heikenwalder, Martin Schneider, Achim Weber, Wilfried Roth, Henning Schulze-Bergkamen, Bruno Christian Koehler

**Affiliations:** 1 National Center for Tumor Diseases, Department of Medical Oncology and Heidelberg University Hospital, Internal Medicine VI, 69120 Heidelberg, Germany; 2 Clinical Cooperation Unit (CCU) Molecular Tumor Pathology, German Cancer Research Center (DKFZ), Heidelberg 69120, Germany; 3 Institute of Pathology, Department of Surgical Pathology, University Hospital Heidelberg, Heidelberg 69120, Germany; 4 Division of Translational Oncology, National Center for Tumor Diseases and German Cancer Research Center (DKFZ), Heidelberg 69120, Germany; 5 Department of General, Visceral and Transplantation Surgery, University of Heidelberg, Heidelberg 69120, Germany; 6 Department of Immunology, Duke University Medical Center, Durham, NC 27710, USA; 7 Institute of Virology, Technische Universität München and Helmholtz Zentrum München, Munich 81675, Germany; 8 Division of Chronic Inflammation and Cancer, German Cancer Research Center (DKFZ), Heidelberg 69120, Germany; 9 Institute of Surgical Pathology, University and University Hospital Zurich, Zurich 8091, Switzerland; 10 Institute of Pathology, University Medical Center Mainz, Mainz 55131, Germany; 11 Department of Internal Medicine II, Marien-Hospital, Wesel 46483, Germany

## Abstract

Colorectal cancer (CRC) is the second most common malignant neoplasia in women and men worldwide. The B-cell lymphoma 2 (Bcl-2) protein family is mainly known for its pivotal role in the regulation of the mitochondrial death pathway. Anti-apoptotic Bcl-2 proteins may provide survival benefits and induce therapy resistance in cancer cells. Among anti-apoptotic Bcl-2 proteins, we found solely Bcl-x_L_ strongly upregulated in human CRC specimens. In order to study protein function in the context of tumor initiation and progression *in vivo*, we generated a mouse model lacking Bcl-x_L_ in intestinal epithelial cells (Bcl-x_L_^IEC-KO^). If challenged in an inflammation-driven tumor model, Bcl-x_L_^IEC-KO^ mice showed a significantly reduced tumor burden with lower tumor numbers per animal and decreased tumor sizes. Analysis of cell death events by immunohistochemistry and immunoblotting revealed a striking increase of apoptosis in Bcl-x_L_-negative tumors. qRT-PCR and immunohistochemistry excluded changes in proliferative capacity and immune cell infiltration as reasons for the reduced tumor load and thereby identify apoptosis as key mechanism. Human CRC tissue was cultured *ex vivo* and treated with the small molecule compound ABT-737, which inhibits Bcl-x_L_ and Bcl-2. Under ABT-737 treatment, the amount of apoptotic tumor cells significantly increased compared with controls, whereas proliferation levels remained unaltered. In summary, our findings identify Bcl-x_L_ as a driver in colorectal tumorigenesis and cancer progression, making it a valuable target for clinical application.

Colorectal cancer (CRC) is one of the most frequently diagnosed cancers throughout the world, with especially high incidences in developed countries. In addition, it is a main cause for cancer-related death in humans.^1^ Despite substantial progress in the development of targeted therapies, patients with metastasized CRC still face a poor prognosis.^2^

The B-cell lymphoma 2 (Bcl-2) protein family is well established for its essential role in the intrinsic apoptotic signaling pathway. Under physiological conditions, pro-apoptotic members like Bax and Bak are sequestered and thereby inhibited by anti-apoptotic relatives like Bcl-x_L_, Bcl-2 or Mcl-1. If apoptotic stimuli like DNA damage or massive protein aggregation occur, then they are sensed by proteins of the BH3-only subgroup, functioning as a molecular switch that determines cells fate. Due to the activity of BH3-only proteins like PUMA and NOXA, pro-apoptotic proteins get released from their binding, leading to subsequent mitochondrial activation and initiation of the downstream apoptosis cascade.^3^ Anti-apoptotic proteins are overexpressed in different tumor entities, supporting cell death avoidance as classical hallmark of cancer.^4, 5^ In CRC, high Bcl-x_L_ expression has been shown to correlate with lower tumor differentiation and poorer overall patient survival.^6^ In contrast, high Bcl-2 levels seem to correlate with a good clinical outcome.^7^

Since anti-apoptotic proteins have always been described as being redundant, with regard to mitochondria activation, the mentioned findings are counterintuitive and underline the necessity of a better understanding of their relevance and commitment in CRC. There is growing evidence that anti-apoptotic proteins have also a role in other cellular processes important for cancer initiation and progression, which might provoke the reported differences. For instance, Mcl-1 has been shown to inhibit cell-cycle progression via binding of proliferating cell nuclear antigen (PCNA)^8^ and Cyclin depending kinase 1 (CDK1).^9^ In addition, it has been implicated in DNA damage repair,^10^ what further enhances the probability of Mcl-1 having a tumor suppressor role besides its cell death-preventing function. In migration and invasion assays, it turned out that Bcl-x_L_, Bcl-2 and Mcl-1 increase the migratory capacity of human CRC cells *in vitro* independent of cell death regulation.^11^

In our study, we aimed at dissecting the role of anti-apoptotic proteins in the context of CRC initiation and progression. Immunohistochemical staining of human CRC samples compared with healthy mucosa identified Bcl-x_L_ as the only anti-apoptotic protein being overexpressed in tumor tissue. In intestine-specific knockout mice, challenged in an inflammation-driven tumor model, the loss of Bcl-x_L_ diminishes carcinogenesis. In addition, we show that elevated Bcl-x_L_ protein levels in human CRC can be therapeutically exploited using an *ex vivo* model. In summary, our findings identify Bcl-x_L_ as a central player in colorectal tumorigenesis and tumor progression, which is an interesting target for clinical application due to its druggability.

## Results

### Bcl-x_L_ is the only anti-apoptotic protein upregulated in human CRC

**Figure 1:**
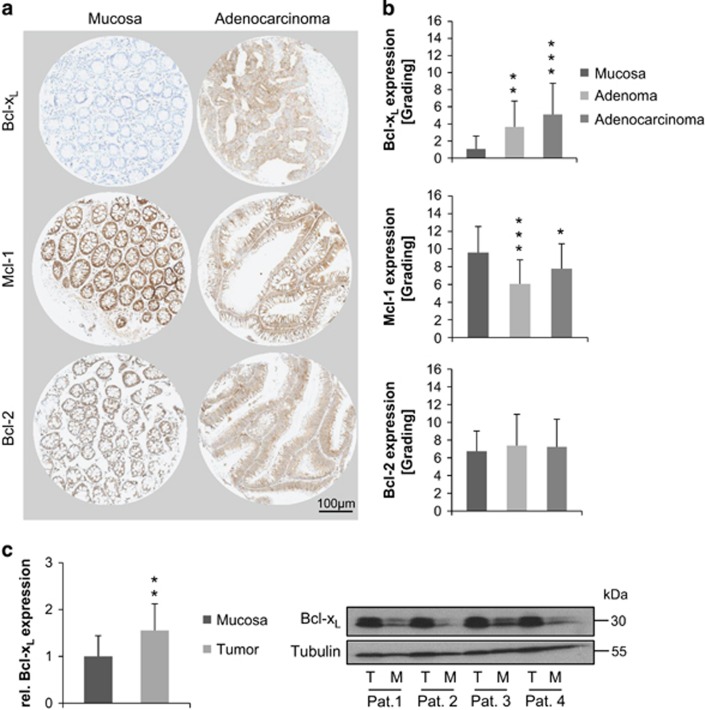
Expression levels of anti-apoptotic Bcl-2 proteins in human CRC. (**a**) IHC staining against Bcl-x_L_, Mcl-1 and Bcl-2 on a TMA, containing normal mucosa (*n*=13), adenoma (*n*=22) and adenocarcinoma tissue (*n*=61). Exemplary spots of mucosal and adenocarcinomal tissue are shown. Scale bar indicates magnification for all panels. (**b**) Evaluation of staining intensities by multiplying values for staining quantity and quality. All *P*-values are calculated using mucosa as control group. Bcl-x_L_ is significantly overexpressed in adenomas (*P*=0.007) and adenocarcinomas (*P*=0.0002), Mcl-1 shows a decreased expression whereas Bcl-2 shows no deregulated expression. (**c**) Significant increase of Bcl-x_L_ expression in CRC tissue compared with the corresponding normal mucosa (*P*=0.01), determined by western blot analysis and subsequent densitometric evaluation (T=Tumor, M=Mucosa, Pat=Patient; *n*=13 patients in total). Exemplary western blot of four patients is shown. Values are expressed as means+S.D. *P<0.05; **P<0.01; ***P<0.001

To investigate the oncogenic role of anti-apoptotic proteins in colorectal carcinogenesis, their expression levels were immunohistochemically analyzed. A tissue microarray (TMA), containing adenoma, adenocarcinoma and normal mucosa specimens was stained for Bcl-x_L_, Mcl-1 and Bcl-2 (Figure 1a). In adenomas, Bcl-x_L_ was found to be significantly overexpressed (*P*=0.007) if compared with normal mucosa with further increase in adenocarcinomal tissue (*P*=0.0002) (Figure 1b). For Bcl-2, no significant differences in the expression levels were found. Surprisingly, Mcl-1 was found to be significantly downregulated in adenomas (*P*=0.0007) with a slight rebound in the malignant stage (*P*=0.03).

Since Bcl-x_L_ expression showed the greatest heterogeneity within the groups, we additionally compared protein expression by western blot analysis in tumor *versus* mucosa samples taken from the same patient. In total, tissue specimens of 13 patients were analyzed. In line with the results obtained from the TMA, a significantly higher expression of Bcl-x_L_ in carcinoma tissue compared with healthy mucosa was observed (*P*=0.01, Figure 1c).

### The knockout of Bcl-x_L_ in intestinal epithelial cells causes no spontaneous phenotype

**Figure 2:**
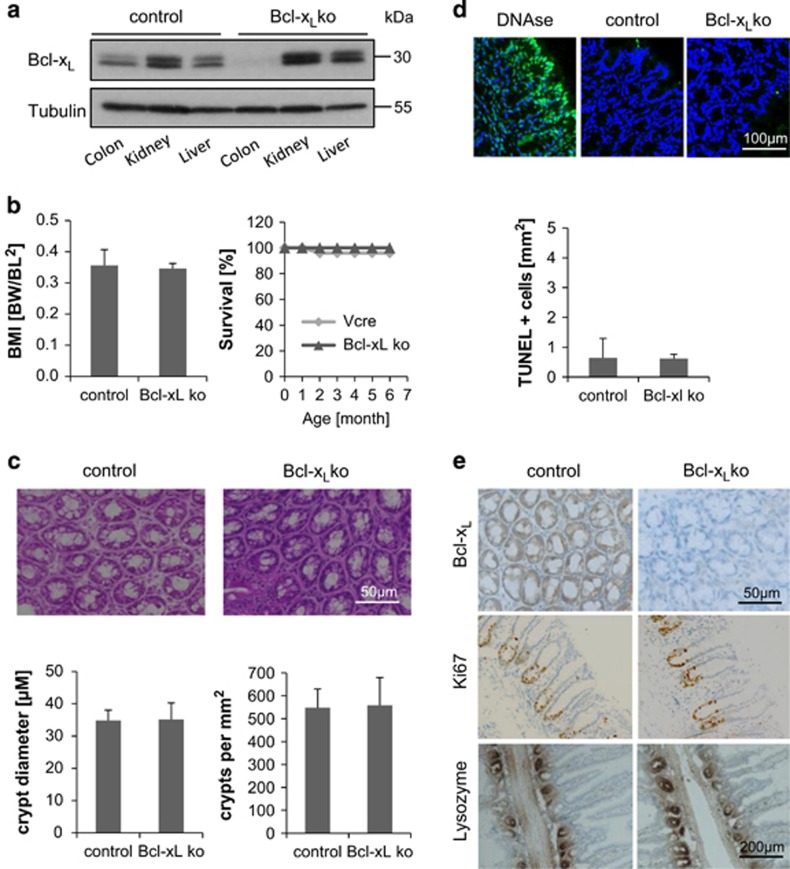
Basal characterization of intestine-specific Bcl-x_L_ knockout mice. (**a**) Western blot analysis of proteins extracted from different tissues of Bcl-x_L_^IEC-KO^ and control mice, proving an organ-specific deletion. (**b**) Bcl-x_L_^IEC-KO^ mice are born healthy, showing no phenotype in terms of BMI or overall survival. (**c**) H&E staining of colonic specimens, revealing the same crypt morphology in Bcl-x_L_^IEC-KO^ and control mice. Scale bar indicates magnification for both pictures. (**d**) Evaluation of cell death rates by TdT-mediated dUTP nick end labeling (TUNEL assay) of fragmented DNA. Except the DNAse-treated positive control, very few TUNEL-positive cells are detectable in colon sections of Bcl-x_L_^IEC-KO^ and control mice. Scale bar indicates magnification for all panels. (**e**) IHC staining of colonic (for Bcl-x_L_ and Ki67 staining) and small intestinal (for lysozyme staining) tissue, showing no differences in proliferation or Paneth cell frequency between Bcl-x_L_^IEC-KO^ and control mice. Upper scale bar indicates magnification for the upper and the middle row, whereas lower scale bar indicates magnification for the lower to pictures. Values are expressed as means+S.D.

Analyses of human CRC tissue revealed Bcl-x_L_ as the only anti-apoptotic protein being overexpressed in the malignant state. To investigate its role for intestinal tissue homeostasis under physiological conditions and for carcinogenesis in an inflammation-driven tumor model, we generated mice with a conditional loss of Bcl-x_L_ in intestinal epithelial cells (Bcl-x_L_^IEC-KO^). The selective loss of Bcl-x_L_ expression in colon lysates of Bcl-x_L_^IEC-KO^ mice was shown by western blot analysis (Figure 2a), proving an organ-specific deletion. Bcl-x_L_^IEC-KO^ mice were born healthy and at expected mendelian ratios. Compared with control littermates, they show no overt phenotype in terms of overall survival and body mass index (BMI) (Figure 2b). For morphometric analysis, crypt diameter and number in H&E-stained colonic crypt sections were determined (Figure 2c) and revealed a normal crypt architecture and morphology. Since the loss of an anti-apoptotic protein might lead to spontaneous cell death induction, a TUNEL (TdT-mediated dUTP-biotin nick end labeling) assay was performed. Compared with the DNAse-treated positive control, neither Cre control nor Bcl-x_L_^IEC-KO^ animals showed a noteworthy amount of TUNEL-positive cells in the colon mucosa (Figure 2d). Immunohistochemical staining of Ki67 as proliferation marker and Lysozyme as marker protein for Paneth cells (Figure 2e) revealed no differences between control and Bcl-x_L_^IEC-KO^ mice in terms of cell-cycle control or Paneth cell function. In summary, there was no basal phenotype in Bcl-x_L_^IEC-KO^ detectable.

### Loss of Bcl-x_L_ inhibits carcinogenesis in an inflammation-driven tumor model

**Figure 3:**
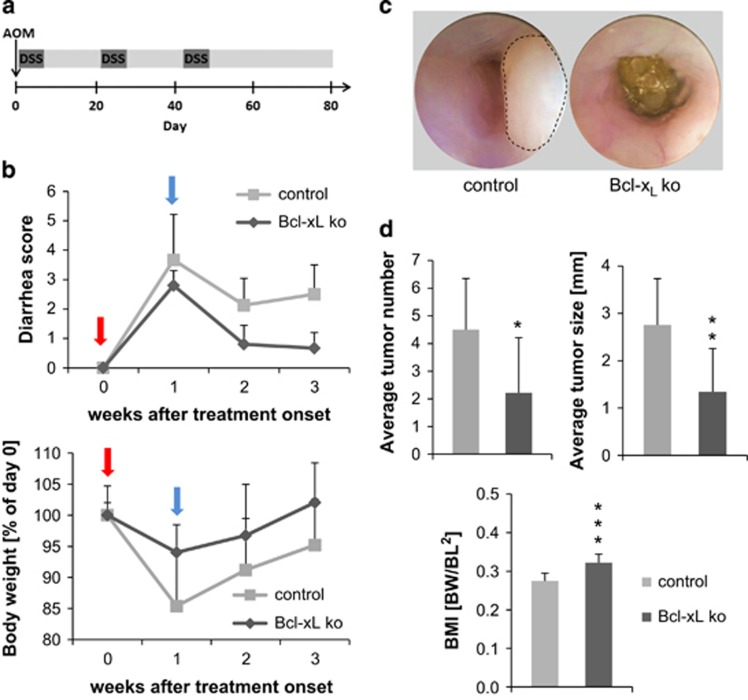
Bcl-x_L_^IEC-KO^ and control mice in an inflammation-driven model for intestinal carcinogenesis. (**a**) Schematic treatment course with intraperitoneal injection of AOM at the start day and three cycles of DSS in the drinking water (2% w/v). (**b**) Diarrhea severity and weight loss after DSS administration (red arrow) and during recovery time (blue arrow). Exemplarily shown for the third DSS cycle. (**c**) Endoscopic images of Bcl-x_L_^IEC-KO^ and control mice. The dashed line shows a neoplastic lesion. (**d**) The determination of tumor number (*P*=0.03) and size (*P*=0.008) at the end of treatment shows a significantly lower tumor burden in Bcl-x_L_^IEC-KO^ compared with control mice. Furthermore, the BMI is higher in Bcl-x_L_^IEC-KO^ mice (*P*=0.0003). Values are expressed as means+S.D. Control mice: *n*=8; Bcl-x_L_^IEC-KO^ mice: *n*=9. *P<0.05; **P<0.01; ***P<0.001

Since our data show that Bcl-x_L_ is markedly upregulated in human CRC tissue (Figure 1), we reasoned that Bcl-x_L_^IEC-KO^ mice could be more resistant to experimentally induced tumorigenesis. Therefore, mice were injected intraperitoneally with the mutagenic agent azoxymethan (AOM) to initiate intestinal tumor formation, which was subsequently promoted by three cycles of the pro-inflammatory reagent dextran sodium sulfate (DSS) in the drinking water (Figure 3a). During the course of treatment, Bcl-x_L_^IEC-KO^ mice showed a better health status mirrored by less severe diarrhea and less pronounced weight loss during DSS cycles (Figure 3b). Rigid colonoscopy of mice 80 days after AOM injection revealed a higher tumor burden in control animals (Figure 3c). The average tumor number (*P*=0.03) and size (*P*=0.008) of single tumors were significantly lower in Bcl-x_L_^IEC-KO^ mice compared with controls (Figure 3d). Furthermore, the BMI was higher in Bcl-x_L_^IEC-KO^ mice at the end of treatment (*P*=0.0003). Taken together, these observations argue for a reduced susceptibility of Bcl-x_L_^IEC-KO^ mice toward chemically induced and inflammation augmented carcinogenesis.

### Tumors of Bcl-x_L_^IEC-KO^ mice show increased cell death without compensatory proliferation

**Figure 4:**
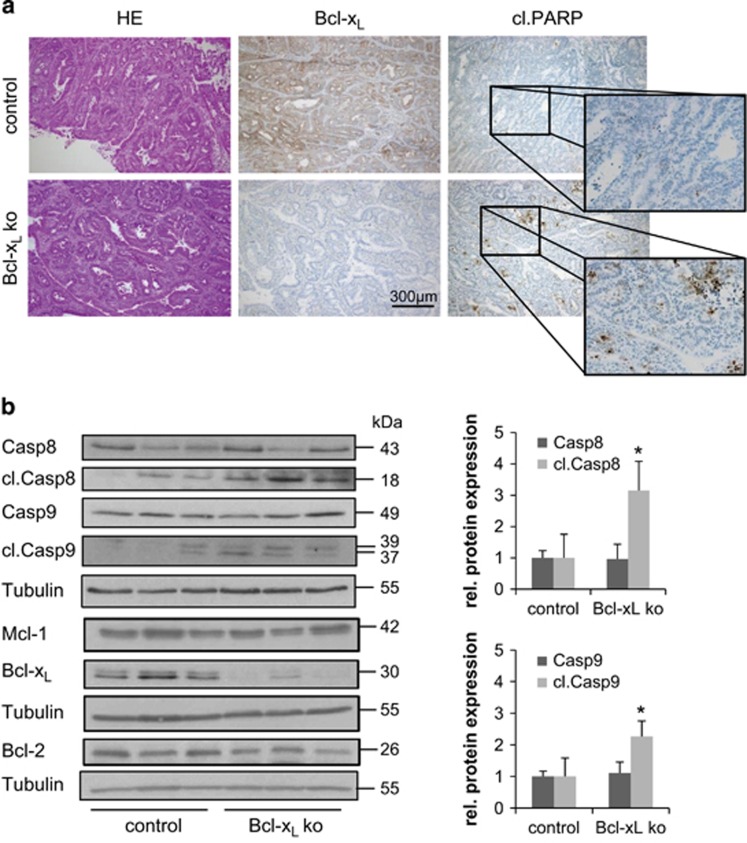
Analysis of cell death events in AOM/DSS-induced tumors. (**a**) H&E staining (left column), identifying gathered neoplastic lesions as well-differentiated adenocarcinomas. IHC staining against Bcl-x_L_ and cleaved PARP, revealing remarkable amounts of apoptotic cells in Bcl-x_L_-negative but not control tumors. (**b**) Immunoblotting and subsequent densitometric analysis, showing a 3.2-fold upregulation of cleaved Caspase 8 (*P*=0.035) and a 2.3-fold upregulation of cleaved Caspase 9 (*P*=0.045) in Bcl-x_L_-negative tumors compared with controls. Expression levels of Mcl-1 and Bcl-2 remain unaltered. Values are expressed as means+S.D. *P<0.05

H&E staining of colonic sections, taken from Bcl-x_L_^IEC-KO^ and control mice after AOM/DSS treatment, identified the gathered neoplastic lesions as being well-differentiated adenocarcinomas with similar morphology in Bcl-x_L_^IEC-KO^ and control animals. With regard to cell death, tumors of Bcl-x_L_^IEC-KO^ mice showed a higher positivity for cleaved PARP arguing for an increased rate of apoptosis in Bcl-x_L_-negative tumors (Figure 4a). Immunoblotting was done in order to characterize the subtype of cell death in tumors of Bcl-x_L_^IEC-KO^ mice. Initiator Caspases 8 and 9 were both found to be activated (Figure 4b). Densitometric analysis revealed that the amount of cleaved Caspase 8 was threefold higher in Bcl-x_L_-negative tumors than in comparable controls (*P*=0.035) and the one of cleaved Caspase 9 was more than doubled (*P*=0.045). In addition, expression levels of Bcl-x_L_ itself, Mcl-1 and Bcl-2 were determined by western blot analysis. This revealed no compensatory upregulation of Mcl-1 or Bcl-2 in tumors derived from Bcl-x_L_^IEC-KO^ mice (Figure 4b). For Mcl-1, this finding was further verified by an immunohistochemical staining, comparing expression levels in both mucosa and tumor tissue derived from Bcl-x_L_^IEC-KO^ and control mice (Supplementary Figure S1a) and on the mRNA level by qRT-PCR analysis (Supplementary Figure S1b).

**Figure 5:**
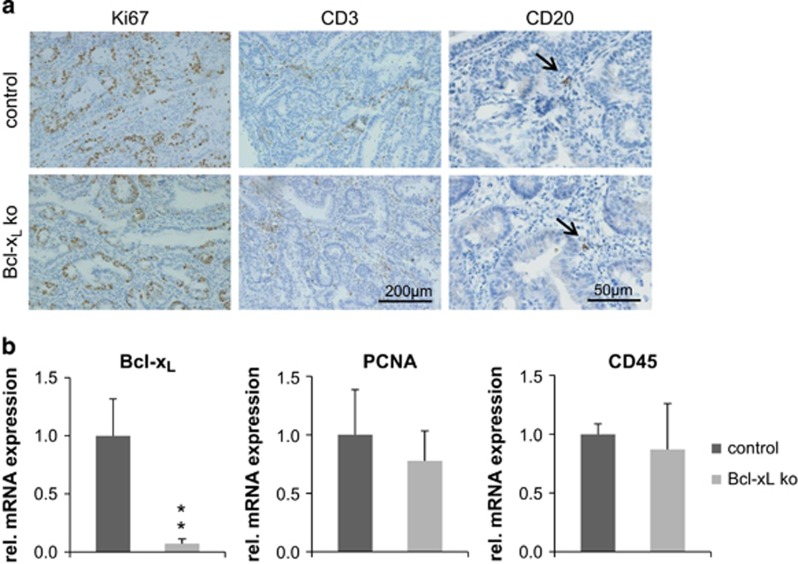
Analysis of proliferation and lymphocyte infiltration in AOM/DSS-induced tumors. (**a**) IHC staining against Ki67 (left column), revealing equal proliferation rates in Bcl-x_L_-negative and control tumors. Staining with antibodies against CD3 (T cells) and CD20 (B cells), showing no differences in T-cell or B-cell (black arrows) abundance in Bcl-x_L_-negative as well as control tumors. (**b**) Determination of Bcl-x_L_, PCNA (proliferation, *P*=0.45) and CD45 (all leukocytes, *P*=0.6) mRNA levels by qRT-PCR, showing no significant differences in the proliferation rate or leukocyte infiltration in Bcl-x_L_-negative and control tumors (*n*=3 per group, measurement done in technical duplicates). Values are expressed as means+S.D. **P<0.01

Ki67 staining revealed that the increase in cell death was not accompanied by increased proliferation in Bcl-x_L_^IEC-KO^ mice (Figure 5a). This observation was further validated by qRT-PCR analysis of RNA extracted from tumor tissue. Here, the relative mRNA levels of PCNA, as an alternative indicator for proliferating cells, were also not significantly changed (Figure 5b). To investigate whether higher lymphocyte infiltration rates might be responsible for the increased activation of Caspase 8 in Bcl-x_L_-negative tumors, immunohistochemical staining was done with antibodies against CD3 to detect T cells and CD20 to detect B cells (Figure 5a). Staining revealed equal abundance of T as well as B cells in Bcl-x_L_-negative and control tumors. Unaltered mRNA levels of the pan-leukocyte marker CD45 further underline this finding (Figure 5b). We conclude that the lower tumor burden found in AOM/DSS-treated Bcl-x_L_^IEC-KO^ mice is not due to a different immune response or proliferation but solely relays on an increased cell death rate.

### The Bcl-x_L_/Bcl-2 inhibitor ABT-737 is effective *in vitro* and *ex vivo*

Since Bcl-x_L_ was found to be overexpressed in human CRC (Figure 1), we sought to exploit the high levels of Bcl-x_L_ using a small molecule inhibitor targeting the protein. We used ABT-737, which is a BH3 mimetic with high affinity to the BH3 groove of Bcl-x_L_ and Bcl-2.^12^

**Figure 6:**
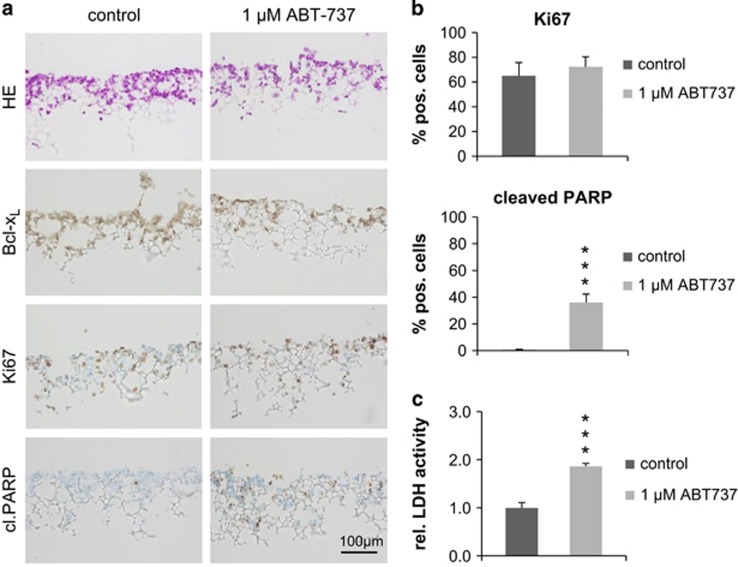
Evaluation of the Bxl-x_L_/Bcl-2 inhibitor ABT-737 in a 3D cell culture approach. (**a**) H&E staining and IHC against Bcl-x_L_, Ki67 and cleaved PARP on scaffold sections, revealing a significant (*P*<0.001) amount of apoptotic HT29 cells in ABT-737 (1 *μ*M for 4 days) treated samples (*n*=5 scaffolds per group). Scale bar indicates magnification for all panels. (**b**) Correlating quantification of Ki67- and cleaved PARP-positive cells, determined by counting, showing a significant increase (*P*<0.001) in dead cells (cleaved PARP) but no significant changes in the proliferative capacity (Ki67) under ABT-737 treatment (*n*=5 scaffolds per group). (**c**) Measurement of LDH in the supernatant of scaffolds after 4 days of treatment with the inhibitor (1 *μ*M), showing a 1.9-fold higher concentration under treatment (*P*<0.001). Values are expressed as means+S.D. ***P<0.001

First, 3D cell culture systems were used for long-term cell culture of human CRC cell line HT29 in a tissue mimicking environment. After 4 days of treatment with ABT-737 (1 *μ*M) or DMSO as control, scaffolds were sectioned and stained for cleaved PARP. Staining showed massive apoptosis induction in ABT-737-treated cells with an average of 36% cleaved PARP-positive cells. By contrast, almost no cell death was detected in DMSO-treated controls (0.5%, *P*<0.001; Figures 6a and b). This observation was validated by measuring lactate dehydrogenase (LDH) in the supernatant of scaffolds as a parameter for tumor cell death. In line with the cleaved PARP staining, LDH activity is almost doubled (1.9-fold; *P*<0.001) in supernatants of ABT-737-treated scaffolds, further substantiating the potency of Bcl-x_L_ inhibition in long-term 3D cell culture (Figure 6c). Furthermore, scaffold sections were stained for Ki67, revealing an unaltered proliferative capacity of HT29 cells under ABT-737 treatment. Expression levels of Bcl-x_L_ itself also remained unchanged in the presence of the inhibitor (Figures 6a and b).

**Figure 7:**
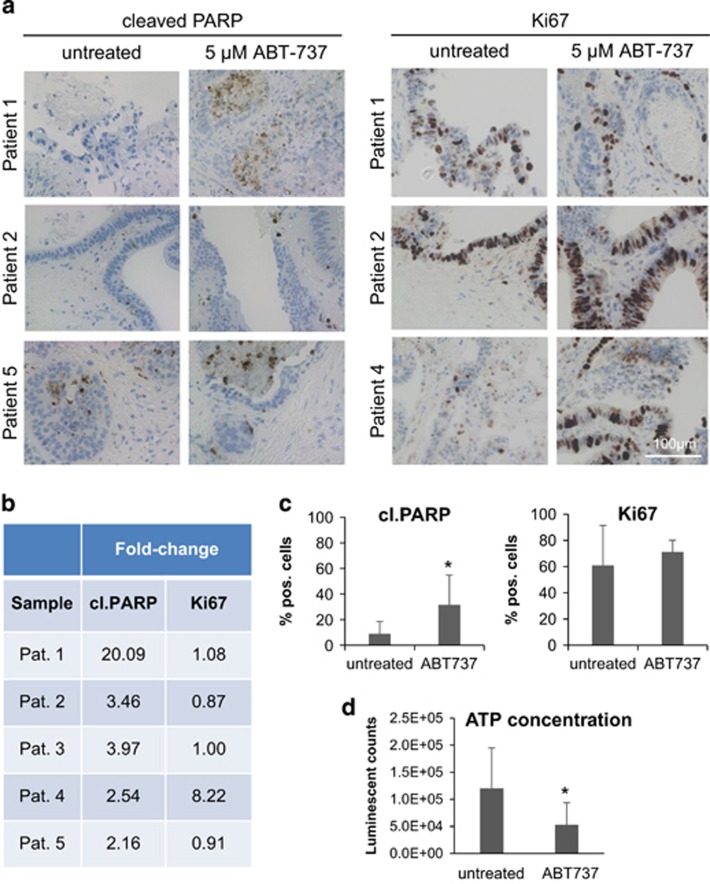
Evaluation of the Bxl-x_L_/Bcl-2 inhibitor ABT-737 in an *ex vivo* tissue culture approach. Human CRC specimens were sliced and kept in culture for 94 h. After 24 h, the medium was supplemented with 5 *μ*M ABT-737 or DMSO as control. (**a**) IHC against cleaved PARP (left-hand side) and Ki67 (right-hand side) on tissue culture sections from three patients. Scale bar indicates magnification for all panels. (**b** and **c**) Table and graphs summarizing changes of cleaved PARP- and Ki67-positive cells, which were determined by counting of control and ABT-737-treated specimens. Quantification revealed a significant increase (*P*=0.028) in dead cells (cleaved PARP) but an unaltered proliferative capacity (Ki67) of ABT-737-treated tumor tissue. (**d**) ATP assay, showing a decreased ATP content in ABT-737-treated tissue specimens (*P*=0.024; *n*=5 scaffolds per group). Values are expressed as means+S.D. *P<0.05

To evaluate the potential of ABT-737 in a human *ex vivo* system, vital CRC specimens of 10 patients were treated with ABT-737 (5 *μ*M) or DMSO for 72 h. After treatment, H&E-stained sections of CRC specimens were assessed for vital tumor cell content and for tissue quality by a pathologist. Thereupon, specimens derived from five patients were further analyzed with regard to cell death and proliferation. Immunohistochemical staining for cleaved PARP revealed a significant increase in the amount of dead cells from 8.9 to 31.5% under ABT-737 treatment (*P*=0.028). Results from a Ki67 staining were in line with the findings obtained in our mouse model and the *in vitro* experiments, showing no significant change in the proliferative capacity of CRC tissue under ABT-737 treatment (Figures 7a–c). Even though, expression levels of Mcl-1 and Bcl-2 seem to be different in individual patients, western blot analysis revealed no significant changes in the expression of anti-apoptotic Bcl-2 proteins under ABT-737 treatment (Supplementary Figure S2). To prove the cell death phenotype, an additional ATP assay was performed. Measured luminescence, which directly correlates with the amount of ATP within the tissue, significantly decreased in ABT-737-treated tissue specimens (*P*=0.024). This argues for a subsidence of tissue viability in presence of the inhibitor (Figure 7d).

## Discussion

Even if anti-apoptotic Bcl-2 proteins have been studied in the context of CRC, the available data are inconsistent and no comprehensive study investigating the therapeutic potential of Bcl-x_L_ is available. Furthermore, no animal models studying the role of Bcl-x_L_ for intestinal pathophysiology including cancer have been generated so far. Thus, we sought to dissect the role of Bcl-x_L_ in human and murine CRC onset and progression with the final aim of testing for a therapeutic value.

Earlier reports by Zhang *et al.*^13^ and Birrocio *et al.*^14^ correlated an upregulation of Bcl-x_L_ with malignant behavior of CRC and a worse clinical course. In line with these studies, we detected an upregulation of Bcl-x_L_ in human CRC compared with healthy mucosa. This observation holds true for a single patient situation and larger cohorts comparing independent healthy mucosa with malignant tissue, arguing for a role of Bcl-x_L_ in human CRC. Birrocio *et al.* identified a significant relationship of high Bcl-x_L_ levels and upregulation of the transcription factor c-MYB. Thus, c-MYB might be a mechanistic link that should be further investigated in future studies.

The counterintuitive downregulation of Mcl-1 in CRC specimens might be due to its unique role in DNA damage repair.^10^ A similar pattern showing a loss of Mcl-1 and an acquirement of Bcl-x_L_ has been described by Krajewska *et al.*^15^ In case of Bcl-2, we did not observe changes in the expression level, indicating a non-redundant and organ-specific function of these anti-apoptotic proteins.

To investigate the role of Bcl-x_L_ for intestinal tissue homeostasis and for pathophysiologic processes like tumorigenesis in further detail, we generated knockout mice lacking Bcl-x_L_ in intestinal epithelial cells. Bcl-x_L_^IEC-KO^ mice showed no overt phenotype in terms of birth rates, growth and survival. Histologic analysis revealed no differences in crypt morphology, cell death rates and proliferation. The lack of a spontaneous phenotype argues for a dispensability of Bcl-x_L_ under normal conditions, which might be due to the *per se* high cellular turnover rates of IECs.^16^ Since we found Bcl-x_L_ being strongly upregulated in human CRC, we supposed that Bcl-x_L_^IEC-KO^ mice could be more resistant to experimentally induced carcinogenesis. The AOM/DSS model is a well-established model for DNA damage induced and inflammation promoted intestinal tumorigenesis.^17^ The lack of Bcl-x_L_ might accelerate apoptosis initiation under unfavorable conditions and thereby prevent chaotic cellular destruction. We hypothesize that in IECs lacking Bcl-x_L_ a swift and immediately executed apoptosis, via a lowered cell death threshold, might prevent greater tissue damage and subsequent mucosal inflammation. This could be the reason for the better health status of Bcl-x_L_^IEC-KO^ mice in terms of diarrhea severity, weight loss and recovery time during the treatment course. The lower tumor burden found in Bcl-x_L_^IEC-KO^ mice underlines the importance of this anti-apoptotic protein for intestinal carcinogenesis. Less tumors in addition to smaller tumor sizes point to a role of Bcl-x_L_ in CRC onset and progression. Immunohistochemical staining showed a remarkable amount of cleaved PARP-positive cells in Bcl-x_L_-negative but not control tumors. Closer analysis of the contributing Caspases revealed activation of Caspase 9 as initiator Caspase of the intrinsic apoptotic pathway as well as activation of Caspase 8 as activator of the extrinsic pathway. Since Bcl-x_L_ prevents permeabilization of the outer mitochondrial membrane, Caspase 9 cleavage is in line with the reported function of the protein.^18^ To investigate whether higher lymphocyte infiltration rates in Bcl-x_L_-negative tumors might be responsible for the Caspase 8 cleavage, immunohistochemical staining and quantitative real-time PCR were done. Even though DSS causes intestinal inflammation, there was no difference in immune cell infiltrates in tumors from Bcl-x_L_^IEC-KO^ and control mice. Instead, our data argue against a strong role of immune cells in the mechanism by which Bcl-x_L_ attenuates malignant transformation. In contrast, cell death appears as the key switch for a lowered tumor burden of Bcl-x_L_^IEC-KO^ mice. The evasion of cell death is a known hallmark of cancer cells and contributes to an aggressive behavior of malignant tissues.^19^ The increased apoptosis in Bcl-x_L_-negative tumors was not accompanied by compensatory accelerated proliferation. We have recently shown that, in contrast to Mcl-1, Bcl-x_L_ has no crucial role in proliferation of CRC cells.^11^ Taken together, our data identify canonical intrinsic apoptosis as the responsible mechanism for the attenuated intestinal tumorigenesis in Bcl-x_L_^IEC-KO^ mice. This is in line with similar observations, made in large B-cell lymphoma, in which low levels of Bcl-x_L_ were associated with high rates of apoptosis.^20^

A variety of small molecules targeting anti-apoptotic proteins (BH3 mimetics) have been designed and tested in clinical trials.^21, 22^ ABT-199 (venetoclax), a Bcl-2-specific inhibitor, has recently been approved by the FDA for treatment of a CLL subtype.^23^ Another BH3 mimetic, ABT-737, was identified by library screening for high-affinity binding of recombinant Bcl-x_L_.^12^ Here, we tested ABT-737 in 3D cell culture systems and human *ex vivo* CRC cultures. We detected a remarkably high induction of cell death among treatment with ABT-737 in both situations, *in vitro* and *ex vivo*. Hence, ABT-737 shows efficacy in CRC treatment in vital human tissue. Even if the clinical development of ABT-737 is ceased due to toxic side effects, the concept of targeting Bcl-x_L_ in CRC retains its value. Recently, it has been demonstrated that KRAS mutations confer apoptosis resistance in CRC via upregulation of Bcl-x_L_ underpinning the role of the protein as a potential target.^24, 25^ Furthermore, Bcl-x_L_ has been identified as a critical survival factor utilizing frequent genomic alterations in a subset of CRCs.^26^

## Conclusions

Here, we identify Bcl-x_L_ as an oncogenic driver in murine and human CRC. Bcl-x_L_ becomes upregulated during the process of cancer onset and progression. Intestine-specific deletion of Bcl-x_L_ renders mice less sensitive toward carcinogenesis, emphasizing the role of Bcl-x_L_ in CRC. Finally, we show that Bcl-x_L_ overexpression in CRC can be therapeutically exploited utilizing BH3 mimetics. In summary, Bcl-x_L_ is a crucial protector from cell death in CRC and needs further attention in clinical trials as a potentially druggable target.

## Materials and Methods

### Human tissues and ethics statement

Specimens of colonic mucosa and primary CRC tissue were taken upon surgical resection in the Department of General and Transplantation Surgery, University of Heidelberg, Germany. The TMA, containing spots of healthy colon mucosa (*n*=13), adenoma tissue (*n*=22) and adenocarcinoma tissue (*n*=61), was obtained from the Tissue Bank of the National Center for Tumor Diseases (NCT, Heidelberg, Germany). The usage of patient tissue for research purposes was approved by the local ethics committee of the University Hospital of Heidelberg. All analyses were done anonymously and written informed content was obtained from all donors.

### Mice

Mice expressing the *Cre*-recombinase under control of the *Villin*-promoter (Villin-Cre) were kindly provided by Dr. W Chamulitrat (Heidelberg, Germany) and mice carrying loxP-flanked alleles of Bcl-x_L_ (Bcl-x_L_^FLOX^) by Prof. Y-W He (Durham, USA). To generate mice with a conditional loss of Bcl-x_L_ in intestinal epithelial cells (Bcl-x_L_^IEC-KO^), Villin-Cre mice were crossbred with Bcl-x_L_^FLOX^ mice. Mice were housed in individually ventilated cages at the SPF animal facility of the University Hospital in Heidelberg, Germany and kept under a 12-h light cycle with *ad libitum* feeding. All experiments on mice were conducted according to Institutional, National and European animal regulations and protocols were approved by local government authorities.

### Immunohistochemistry

Paraffin-embedded TMA and tissue sections were dewaxed and rehydrated using xylene and a series of graded alcohols, followed by heat-induced antigen retrieval with citrate buffer (pH 6). Subsequently, staining was performed by using the NovoLink Polymer Detection System (Leica Microsystems, Wetzlar, Germany), according to the manufacturer's protocol. The following primary antibodies have been used: Bcl-x_L_ (Cell Signaling, Danvers, MA, USA), Bcl-2 (LSBio, Seattle, WA, USA) and Mcl-1 (Sigma, St. Louis, MO, USA). The immunoreactive score (IRS), ranging from 0 to 12, was determined by two independent and experienced examiners. First, separate scores for staining quantity (0–10%=1, 11–50%=2, 51–80%=3, 81–100%=4) and staining quality (unstained=0, weak=1, moderate=2, strong=3) were determined. In the end, the final IRS was calculated by multiplying the two values obtained from the intensity score and the quantity score.^27^ Negative controls were generated by omitting the primary antibody.

Mouse colon tissue (Figures 2e,4a and 5a) was isolated, rinsed with PBS, covered with OCT mounting medium (Science Services, Munich, Germany) and gradually frozen in the gas phase of liquid nitrogen. In all, 8 *μ*m cryosections were cut (Cryostat, Thermo) and fixed in 4% paraformaldehyde (PFA). Antigen retrieval and staining were performed as described. The following primary antibodies have been used for murine tissues: Bcl-x_L_ (Cell Signaling), Ki67, Lysozyme, cleaved PARP, CD3 (all from Abcam, Cambridge, UK) and CD20 (Thermo Fisher, Waltham, MA, USA).

### Protein isolation, SDS-PAGE and western blot analysis

Deeply frozen tissue specimens in RLT buffer (Qiagen, Venlo, The Netherlands) were lysed by using the Precellys Homogenizer 24 (Bertin Technologies, Montigny-le-Bretonneux, France). For further steps, the AllPrep DNA/RNA/Protein Mini Kit (Qiagen) has been used and proteins were isolated from the supernatant according to the manufacturer's instructions. Equal amounts of protein were separated by 12% SDS-PAGE and blotted onto nitrocellulose membranes by standard procedures. Immunoblotting was performed using the following primary antibodies: Bcl-xL, Caspase/cleaved Caspase 8, Caspase/cleaved Caspase 9 (all from Cell Signaling) and Tubulin (Sigma) as well as peroxidase-conjugated secondary antibodies (Santa Cruz Biotechnology, Heidelberg, Germany). Bound antibody was visualized using an enhanced chemiluminescence detection system (Perkin-Elmer, Zaventem, Belgium) and signal intensity was measured using ImageJ (by Wayne Rasband, National Institutes of Health, USA) software.

### AOM/DSS model and mouse endoscopy

Ten-week-old mice (*n*=10 per group) with a body weight of >20 g were injected intraperitoneally with AOM (10 mg per kg body weight). Experimental groups were similar with regard to age and sex ratio.

The mutagenic agent AOM (Sigma) initiates intestinal tumor formation, which is promoted by three cycles of the pro-inflammatory reagent DSS (MP Biomedicals, Santa Ana, CA, USA) in the drinking water (2% w/v). Each cycle lasted 7 days with 14 days of recovery in between (Figure 3a). For evaluation of diarrhea severity, the following score was used: (0) no diarrhea: solid stool with no sign of soiling around the anus. (1) Mild diarrhea: formed stool that appears moist on the outside. Some signs of soiling around anus. (2) Diarrhea: unformed stool with a mucous-like appearance. Considerable soiling around the anus. (3) Severe diarrhea: mostly clear or mucous-like liquid stool with very minimal solid present and considerable soiling around anus. (4) Bloody diarrhea: severe diarrhea with bloody contingent and considerable soiling around anus.

High-resolution mouse endoscopy was performed as described^28^ with a Mainz COLOVIEW endoscopic system (Karl Storz, Tuttlingen, Germany). In all, 5% isoflurane in oxygen was administered for anesthesia initiation and then decreased to 2% in oxygen for anesthesia maintenance. Eighty days after injection, mice were killed by cervical dislocation and bowel cavity was opened. The colon was removed, rinsed with PBS and opened longitudinally. Colorectal tumors were counted and tumor diameters were measured with a sliding caliper. Some tumors were taken for immunohistochemical analyses, whereas others were used for protein isolation as described.

### TUNEL assay

To detect apoptotic enterocytes, a TUNEL assay was performed. Therefore, mouse colon tissue was isolated, rinsed with PBS, transferred in OCT mounting medium (Science Services, Munich, Germany) and gradually frozen in the gas phase of liquid nitrogen. In all, 8 *μ*m cryosections were stained by using the ‘*In Situ* Cell Death Detection Kit, Fluorescein' (Roche Diagnostics, Risch, Switzerland), according to the manufacturer's instructions. TUNEL-stained specimens were imaged with a fluorescence microscope, using a 488 nm excitation laser with emission at 530 nm.

### RNA extraction and qRT-PCR analysis

Total RNA was extracted from murine tissues by using the AllPrep DNA/RNA/Protein Mini Kit (Qiagen), according to manufacturer's instructions. In all, 1 *μ*g of total RNA was reverse transcribed in a final volume of 20 *μ*l using random primers as previously described.^29^ qRT-PCR was performed using primer assay kits (Qiagen) and the LightCycler480 software package (Roche, Mannheim, Germany). Each sample was run in technical duplicates, and mRNA expression was normalized to the mRNA level of GAPDH.

### Cell line and 3D cell culture

The human CRC cell line HT29 was purchased from ATCC (Manassas, VA, USA). Cells were maintained in RPMI+GlutaMAX (Gibco, Karlsruhe, Germany) supplemented with 10% heat-inactivated fetal calf serum (PAA Laboratories, Colbe, Germany), 1% penicillin/streptomycin (PAA Laboratories), 1% HEPES (Gibco) and 1% non-essential amino acids (Gibco) and cultured in a humidified atmosphere (37 °C, 5% CO_2_). The cells were regularly tested for contaminations and routinely subcultured twice a week.

To grow human HT29 CRC cells in a three-dimensional culture assay, Alvetex scaffolds (Reinnervate, Sedgefield, UK) were used. Seeding of cells was done as described previously.^11^ After 24 h, medium was changed and additionally supplemented with 1 *μ*M ABT-737 (Selleckchem, Munich, Germany) or DMSO as control. After 4 days of treatment, in which medium and drug were renewed every second day, scaffolds were harvested, cryosectioned and immunostained as described.^11^ For staining, a primary antibody against cleaved PARP (Abcam) and the NovoLink Polymer Detection System (Leica Microsystems) have been used, according to the manufacturer's protocol. At least 10 pictures per section were captured with an inverted microscope (Keyence, Neu-Isenburg, Germany), and the number of cleaved PARP-positive cells was determined by manual counting.

### Tissue culture

Tumor tissue from 10 patients with CRC was collected upon surgical resection of the primary tumor. Tumor tissue was cut into 300 *μ*m thick slices as described,^27, 30^ transferred onto filter membrane inserts and placed in culture medium (DMEM supplemented with penicillin: 100 U/ml and streptomycin: 100 mg/ml) containing six-well plates. Tissue specimens were kept at the air–liquid interface for up to 94 h. After 24 h of incubation in medium, cancer specimens were treated with the small molecule inhibitor ABT-737 (2.5 and 5 *μ*M) or the respective vector substance (DMSO) for 72 h, by supplementing the culture medium with the mentioned compounds. Finally, tissue slices were either used for performing an ATP assay or fixed in 10% formalin and paraffin-embedded. In all, 4 *μ*m sections were immunohistochemically stained with antibodies against cleaved PARP and Ki67.

### ATP assay

In all, 10 mg frozen human colon carcinoma tissue were homogenized (Bioruptor sonication system, Diagenode, Seraing, Belgium) in 80 *μ*l CellTiter-Glo buffer of the CellTiter-Glo Luminescent Cell Viability Assay (Promega, Madison, WI, USA) for 30 min. Tissue lysate was centrifuged at 14 000 r.p.m. for 10 min at 4 °C to spin down cellular debris. The supernatant was analyzed according to the instructions provided by the manufacturer. The luminescent signal was recorded 10 min after reagent addition (Victor X3 Multimode Plate Reader, Perkin-Elmer, Baesweiler, Germany).

### Statistical analysis

The Student's *t*-test was used to analyze data obtained in the 3D-scaffold (unpaired, two-sided) and tissue culture (paired, two-sided) experiments. In the evaluation of all other data, significant differences were identified by using the Mann–Whitney *U*-test. R 3.1.3 statistic software was used for all statistical analyses (www.R-project.org). *P*-values <0.05 were considered as significant and are indicated as following: *P<0.05, **P<0.01, ***P<0.001.

## Abbreviations

AOM: azoxymethan
Bak: Bcl-2 homologous antagonist/killer
Bax: Bcl-2-associated X protein
Bcl-2: B-cell lymphoma 2
Bcl-x_L_: B-cell lymphoma-extra large
Bcl-x_L_^IEC-KO^: Mice lacking Bcl-x_L_ in intestinal epithelial cells
BH3: Bcl-2 homology domain 3
CRC: colorectal cancer
DSS: dextran sodium sulfate
IECs: intestinal epithelial cells
Mcl-1: myeloid cell leukemia 1
PARP: poly-(ADP-ribose)-polymerase
qRT-PCR: quantitative real-time polymerase chain reaction
