# Supplementary figures and images for: Bcl-xL is an oncogenic driver in colorectal cancer
Source: Cell Death Dis. 2016 Aug 18;7(8):e2342–. doi: 10.1038/cddis.2016.233 (PMC5108319; doi:10.1038/cddis.2016.233)

a

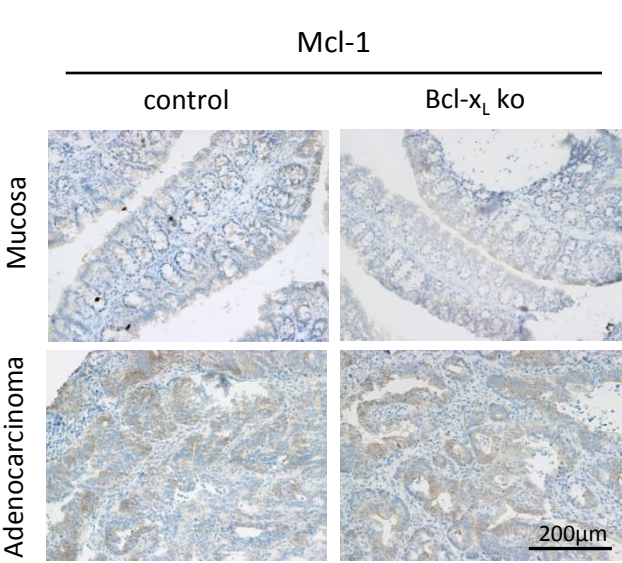

b

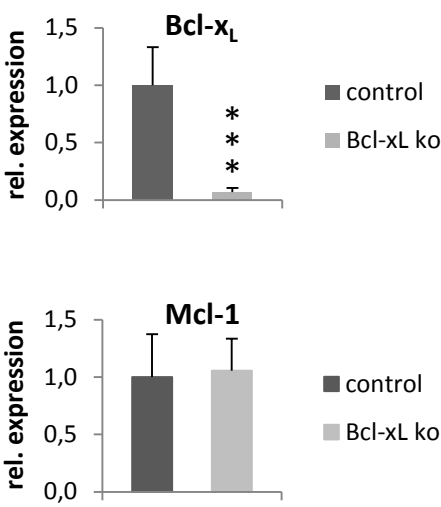

Supplement: Supplementary Figure S1 [file cddis2016233x1.pdf]

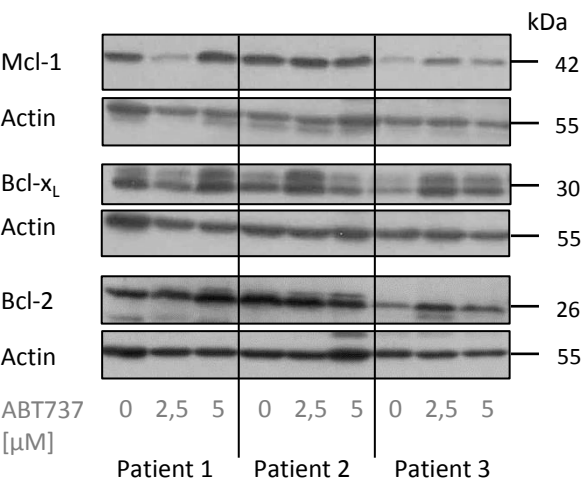

Supplement: Supplementary Figure S2 [file cddis2016233x2.pdf]
