# Supplementary material for: Bcl-xL is an oncogenic driver in colorectal cancer
Source: Cell Death Dis. 2016 Aug 18;7(8):e2342–. doi: 10.1038/cddis.2016.233 (PMC5108319; doi:10.1038/cddis.2016.233)
Supplement: Supplementary Figure Legends [file cddis2016233x3.doc]

**Supplementary Figure Legends**

*Figure S1: Expression levels of Mcl-1 in the intestine of Bcl-x_L_^IEC-KO^ and control mice*. **a)** IHC against Mcl-1, comparing expression levels in both mucosa and tumor tissue derived from Bcl-x_L_^IEC-KO^ and control mice (n=5 mice per group). **b)** Determination of Bcl-x_L_ and Mcl-1 mRNA levels by qRT-PCR, showing no significant differences in the expression of Mcl-1 in Bcl-x_L_ negative and control tumors. (n=3 per group, measurement done in technical duplicates). Values are expressed as means + SD. **p < 0.001.

*Figure S2:* *Expression levels of anti-apoptotic Bcl-2 proteins in human CRC specimens under ABT-737 treatment.* Western Blot analysis, revealing no significant changes in the expression of anti-apoptotic Bcl-2 proteins under ABT-737 treatment (2.5 µM and 5µM or DMSO as control for 72h).
